# How Self-Efficacy toward, Perceived Importance of, and Beliefs about Smoking Cessation Support Impact-Related Behaviors in Japanese Nursing Professionals

**DOI:** 10.3390/ijerph19042304

**Published:** 2022-02-17

**Authors:** Izumi Sezai, Chie Taniguchi, Ituro Yoshimi, Tomoyasu Hirano, Fumihiko Wakao

**Affiliations:** 1 Community Health Nursing Section, National Defense Medical College, Tokorozawa 359-8513, Japan; 2 Institute for Cancer Control, National Cancer Center, Tokyo 104-0045, Japan; amachi@kej.biglobe.ne.jp (C.T.); iyoshimi@ncc.go.jp (I.Y.); tohirano@ncc.go.jp (T.H.); fwakao@ncc.go.jp (F.W.); 3 Chronic Illness Care Nursing, College of Nursing, Aichi Medical University, Nagakute 480-1195, Japan

**Keywords:** nurses, smoking cessation support, structural equation modeling, Japan

## Abstract

This study investigated the relationships among Japanese nursing professionals’ percetions of the importance of smoking cessation support (SCS), attitude toward SCS, SCS self-efficacy, and SCS behaviors. An anonymous, self-administered questionnaire was administered to 613 nursing professionals (valid response rate: 89.9%) who participated in SCS workshops in Japan between May 2019 and February 2020. The survey measured factors such as SCS behaviors (the 5 As) and attitude toward SCS. Participants responded that they “always” or “usually” performed the 5 As at the following rates: Ask, 65.6%; Advise, 46.7%; Assess, 34.4%; Assist, 19.7%; and Arrange, 20.9%. Significant differences in implementation rates between “non-engagers” and “engagers” were found for all steps except Ask. Those who engaged daily in SCS had significantly higher scores for SCS behaviors and SCS perceived importance, attitude, and self-efficacy than those who did not. Structural equation modeling yielded a model with 61% explanatory power, which demonstrated that beliefs about and perceived importance of SCS had a greater impact on SCS behaviors than self-efficacy. Promotion of SCS behaviors among nursing professionals in Japan requires the beliefs about and recognition of the importance of SCS to be improved. The importance of engaging in SCS daily is also recommended.

## 1. Introduction

Smoking is a major contributor to death from non-communicable chronic diseases; addressing this problem is an important part of the public health agenda [1]. Smoking cessation has been proven to reduce the risk of death from cardiovascular disease [2], chronic obstructive pulmonary disease [3], and cancer [4].

Nurses make up the largest proportion of health care workers [5], and they are in a position to support smoking cessation, not only in medical institutions, but also in various health and welfare settings in the community [6]. Nurses are considered to be more effective as the first line of cessation support because they spend the most time with patients [7]. The International Council of Nurses, in collaboration with other national organizations, also encourages member organizations to make efforts to bring government and public attention to the harmful effects of tobacco on health and to encourage governments to reduce, curb, and eliminate tobacco use, including providing access to smoking cessation programs [8].

Regarding the effectiveness of nursing interventions for smoking cessation support (SCS), the Agency for Health Care Research and Quality Clinical Practice Guideline (AHRQ) reports that smoking cessation advice from nurses may increase smoking cessation rates [9]. A meta-analysis by the Cochrane Collaboration reported that advice and support from nurses may increase the success rate of SCS, both in hospital and community settings [10]. Overall, nursing SCS plays an important role in helping patients to successfully quit smoking. However, nurses’ SCS have not always produced satisfactory results [11,12,13].

Self-efficacy and willpower have been associated as facilitators for nurses’ SCS; Devries et al. [14] found that it is the intention to act that is directly related to behavior, and that intention is influenced by self-efficacy for the behavior, attitudes toward the behavior, and subjective norms. Based on this theoretical background, Bolman et al. [15] concluded that the factors that lead to SCS among nurses are the need to increase their attitude and self-efficacy toward smoking cessation support. These elements have also been found to influence medical nurses’ compliance with SCS guidelines [16]. A review by Thornberry et al. identified factors that promote SCS by occupational health nurses; it found that the factors that contributed to the implementation of SCS among nurses were attitude, innovativeness, perceived social impact, and self-efficacy [17]. It is also important to have an attitude that strongly encourages nurses to quit smoking. Attitudes toward smoking cessation were shown to be positively correlated with the components of smoking cessation counselling, termed the 5 As. This result indicates the need to encourage and provide opportunities for nurses to receive training on SCS [11].

A previous cohort study in Japan reported the importance of counseling by nurses in maintaining patients’ self-efficacy to quit smoking and helping them achieve smoking cessation [18]. A study by Li et al. has revealed the interrelationship of these factors, in the context of SCS interventions, by public health nurses for pregnant women about to give birth [19]. This study was conducted in a cohort of only public health nurses. However, to date, no study has examined the extent to which psychological factors (such as self-efficacy and attitudes towards SCS) relate to improving motivation for SCS among various types of nurses in Japan and to what extent they are structurally related.

The purpose of this study is to structurally examine the relationship between the perceptions of importance, attitudes, and self-efficacy toward SCS and SCS from Japanese nursing professionals in all settings. This will be useful in examining factors that may further promote SCS provided by nursing professionals.

The research questions for this study are as follows (within the nursing profession in Japan):

(1) To ascertain whether self-efficacy for SCS, and perceptions and attitudes of the importance of SCS influence the implementation of SCS.
(2) To structurally clarify whether self-efficacy for SCS, or perceptions and attitudes of the importance of SCS have a stronger influence on the implementation of SCS.

## 2. Materials and Methods

### 2.1. Participants

From May 2019 to February 2020, 682 nursing professionals (public health nurses, midwives, nurse practitioners, and practical nurses) participated in SCS workshops that were held specifically for nursing professionals in 13 prefectures of Japan by the prefectural nursing associations. The participants were the 613 nursing professionals who provided a valid survey response (valid response rate: 89.9%).

Details of the study participants are in Table 1. The numbers of workshop participants for each prefectural nursing association were as follows: Yamagata Prefecture, 51; Kumamoto Prefecture, 71; Tochigi Prefecture, 25; Chiba Prefecture, 27; Gunma Prefecture, 25; Hokkaido Prefecture, 71; Yamanashi Prefecture, 50; Osaka Prefecture, 82; Kyoto Prefecture, 37; Okinawa Prefecture, 67; Shimane Prefecture, 35; Saitama Prefecture, 119; and Aichi Prefecture, 22.

### 2.2. Evaluation Method and Survey Content

Anonymous, self-administered questionnaire surveys were conducted before and after the workshops. The content of the questionnaire was reviewed by several fellows of the Japan Society for Tobacco Control, based on previous studies [12,20,21]. The data were taken from the results before the training session in order to quantitatively analyze current perceptions, excluding the influence of the educational program.The surveys included the following aspects [Table 2]:

I. Basic attributes.
II. Sex, age, years as a nursing professional, smoking status, type of nurse, managerial position (yes/no), type of facility in which they work, whether they routinely engage in SCS.
III. SCS behaviors.

Questions regarding SCS behaviors were 1–6 in Table 2.

IV. SCS self-efficacy.

Questions regarding SCS self-efficacy were 7–9 in Table 2.

V. Perceived importance of and attitude toward SCS (Importance).

Questions regarding perceived importance of and attitude toward SCS were 10–19 in Table 2.

### 2.3. Ethical Considerations

The nursing associations and participants were provided oral explanation of the following points at the time of the survey and written explanation on the questionnaire form: Survey participation would be voluntary, there would be no consequences for the answers provided, the names of individuals or facilities will be anonymized, and answering the questionnaire constituted consent to participation in a study concerning evaluation of the workshop. This study was approved by the research ethics review committee of the National Cancer Center of Japan (Notice No. 6000-005, 25 December 2018).

### 2.4. Statistical Analysis

Basic attributes, SCS behaviors, SCS self-efficacy, perceived importance of SCS, and attitude toward SCS were expected to differ depending on whether the respondent had the opportunity to engage daily with SCS for patients or clients. Analyses were therefore conducted once for all participants and again after dividing the participants into those who were already regularly engaging in SCS and those who were not.

Intergroup categorical and numerical data were participants to univariate analysis with the Mann–Whitney *U* test or chi-squared (χ^2^) test. Next, the results of exploratory factor analysis of the survey items were used to name latent variables and establish a structural model, which was then examined with structural equation modeling.

Structural equation modeling was repeated while monitoring path directions, standardized estimates, χ^2^ values, comparative fit index (CFI), adjusted goodness of fit index (AGFI), and root mean square error of approximation (RMSEA) to find the optimal model. The adoption criteria for the hypothetical model’s goodness of fit were as follows: CFI and AGFI of ≥0.9 and RMSEA of ≤0.05. Statistical significance was defined as a test statistic of *p* < 0.05 and a critical ratio (CR) absolute value of ≥1.96.

Analyses were performed using IBM SPSS or AMOS Ver. 25 (IBM Corp., Armonk, NY, USA). Missing values in the survey data were excluded on an item-by-item basis before aggregation and analysis.

## 3. Results

### 3.1. Basic Attributes

Table 1 presents the basic attributes of the study participants. The mean age (standard deviation in parentheses) was 44.2 years (10.6), and the mean number of years as a nursing professional was 19.6 years (10.3). Concerning occupation, 79.8% of the participants were nurse practitioners, and 14.8% were public health nurses. The participants were primarily employed in hospitals (71.9%), followed by medical offices or clinics (7.8%).

Before the workshops, 266 participants (43.4%) responded that they regularly engaged with SCS (hereafter, “engagers”), and 347 (56.6%) responded that they did not (hereafter, “non-engagers”). The basic attributes that differed between these groups were sex, smoking status, type of nurse, managerial position, and type of facility. However, the effect size for all items was less than 0.3.

### 3.2. Comparison of the Perceived Importance of SCS, Attitude toward SCS, Self-Efficacy Related to SCS, and SCS Behaviors 

Table 2 shows the results of the score analysis for perceived importance, attitude, self-efficacy, and behaviors of all the participants, engagers, and non-engagers. Engagers were significantly more likely than non-engagers to do all of the SCS behaviors except “Ask”. Additionally, items related to self-efficacy, importance, and beliefs about SCS scored significantly higher for engagers than for non-engagers on many items. The Items that showed an effect size of 0.3 or more were No. 3.4.5.7.8.

### 3.3. Exploratory Factor Analysis 

Exploratory factor analysis (maximum likelihood method, promax oblique rotation) was performed to identify latent variables for structural equation modeling. The observed variables consisted of a total of 20 items: six items on SCS behaviors, three items on SCS self-efficacy, ten items on attitudes and perceived importance of SCS, and, with reference to the results of Li et al., years as a nursing professional [19]. The analysis identified the four factors shown in Table 3: Factor 1 [smoking cessation support behaviors], Factor 2 [smoking cessation support self-efficacy], Factor 3 [importance of smoking cessation support], and Factor 4 [beliefs about smoking cessation support] (hereafter, square brackets represent latent variables). Cronbach’s α coefficients for the extracted factors were 0.833, 0.479, 0.739, and 0.634, respectively.

### 3.4. Structural Equation Modeling

**Figure 1:**
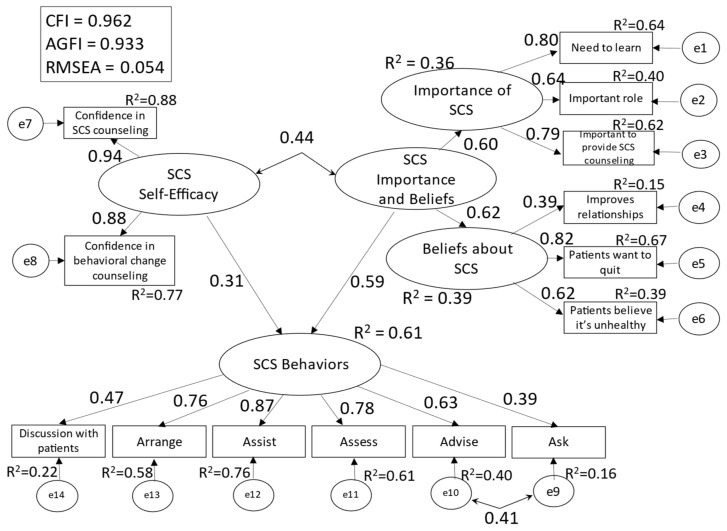
Conceptual Model 2 (all participants).

**Figure 2:**
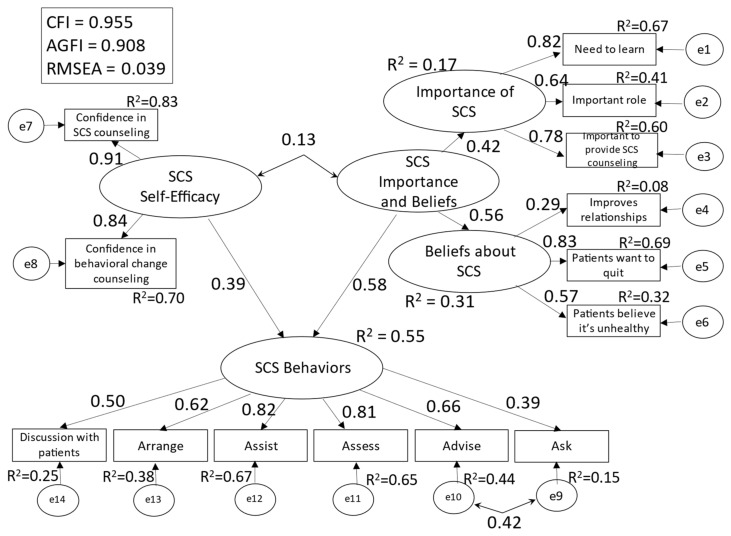
Conceptual Model 2 (SCS non-engagers).

Conceptual models were created with reference to the four latent factors identified in the exploratory factor analysis (Figure 1 and Figure 2). Six items found to have a factor loading of 0.3 or below in factor analysis were excluded from the modeling process: 9. Value of SCS counseling; 10. Importance of smoking cessation for (their own) patients or clients; 11. Perceived importance of SCS at their organization; 14. (Asking patients or clients about smoking) increases the chance of smoking cessation; 19. Smoking cessation counseling is time-consuming; and years as a nursing professional. The validity of the conceptual models was compared using goodness of fit indices.

Model 1 (all participants), in which each of the four latent factors except the first one had an effect on the first factor [SCS behavior], showed solution convergence, but with an inadequate goodness of fit (CFI = 0.962, AGFI = 0.933, RMSEA = 0.055). Moreover, the multiple correlation coefficient (hereafter, R^2^) for [SCS behaviors] was 0.46. Model 2 (all participants) (Figure 1), which consolidated [importance of SCS] and [beliefs about SCS] into [SCS importance and beliefs], showed satisfactory goodness of fit (CFI = 0.962, AGFI = 0.933, RMSEA = 0.054). Further, the R^2^ (0.61) for [SCS behaviors] was higher in Model 2 than in Model 1. Thus, Model 2 was adopted.

**Figure 3:**
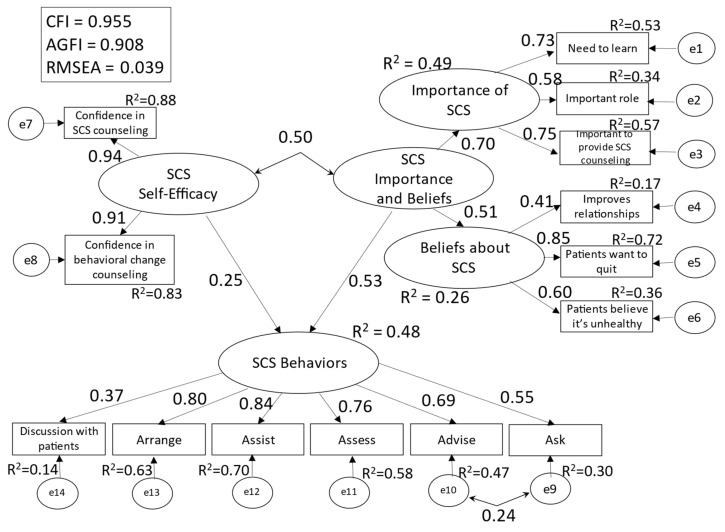
Conceptual Model 2 (SCS engagers).

The results of multigroup structural equation modeling of Model 2 for engagers and non-engagers are shown in Figure 2 and Figure 3.

The model’s goodness of fit indices were as follows: CFI = 0.955, AGFI = 0.908, and RMSEA = 0.039. The R^2^ values for [SCS behaviors] were 0.55 for non-engagers and 0.48 for engagers. The effect size was 1.96 (strong) for SCS self-efficacy and 0.73 (strong) for SCS importance/belief in non-engagers, and 0.29 (moderate) for SCS self-efficacy and 0.38 (strong) for SCS importance/belief in engagers.

For non-engagers, the path coefficient (hereafter, β) between [SCS self-efficacy] and [SCS behaviors] was 0.39, and β between [SCS importance and beliefs] and [SCS behaviors] was 0.58. The covariance between [SCS self-efficacy] and [SCS importance and beliefs] was 0.13 (non-significant difference). For engagers, the β between [SCS self-efficacy] and [SCS behaviors] was 0.25, and β between [SCS importance and beliefs] and [SCS behaviors] was 0.53. The covariance between [SCS self-efficacy] and [SCS importance and beliefs] was 0.50. Pairwise comparison of non-engagers and engagers only showed a significant difference in the covariance between [SCS self-efficacy] and [SCS importance and beliefs] (CR = 2.447).

Among the observed variables of [SCS behaviors], the items strongly explained by [SCS self-efficacy] and [SCS importance and beliefs] (R^2^ > 0.5) were “Assist” (R^2^ = 0.67) and “Assess” (R^2^ = 0.65)” for non-engagers, and “Assist” (R^2^ = 0.70), “Arrange” (R^2^ = 0.63), and “Assess” (R^2^ = 0.58) for engagers.

## 4. Discussion

Using structural equation modeling, this study quantitatively analyzed the extent to which nursing professionals’ SCS self-efficacy, perceived importance, and beliefs explain their SCS behaviors. One instructive finding was that self-efficacy related to SCS, personal perception of SCS as important, and beliefs about SCS may facilitate nursing professionals’ SCS behaviors. 

In Model 2, the R^2^ values for [SCS behaviors] in Figure 1, Figure 2 and Figure 3 show the validity of the model with the said factors. Explanatory strength did not differ between participants who engaged daily with SCS and those who did not, confirming that perceived importance and beliefs about SCS may be greater facilitators of SCS behaviors than self-efficacy. Accordingly, nursing professionals’ performance of SCS behaviors requires not only heightened self-efficacy but also an understanding that smoking cessation is necessary for all patients, that nursing professionals play a major role in the smoking cessation process, and that they should not hesitate to discuss smoking cessation with their patients as doing so improves the patient–nurse relationship. 

Choi et al. measured the predictors of intention to practice SCS among nurses and their explanatory power [22]. They found that intention to practice SCS consisted of perceived barriers to SCS (0.13), willingness to receive SCS training (0.12), positive attitude toward SCS (0.20), positive support from workplace and colleagues (0.30), and self-efficacy related to SCS (0.15), which explained 45% of the model’s variance. Although these factors resemble the facilitators of SCS intervention in the present study, Choi et al.’s study did not measure actual SCS behaviors. The results of this study are considered novel findings. Li et al. reported that self-efficacy (β = 0.52) influences Japanese public health nurses’ SCS behaviors toward pregnant women (R^2^ = 0.41) [19]. Further, Chatdokmaiprai et al. reported that self-efficacy (β = 0.51) impacts occupational health nurses’ SCS behaviors (coefficient of determination 38%) and that nursing professionals’ attitude toward SCS (β = 0.37) in turn influences self-efficacy [23]. The present study largely supports these findings. At the same time, Li et al.’s model found that public health nurses’ years of experience positively influenced self-efficacy, but years of nursing experience were not found to be a significant factor in the present study [19]; Chatdokmaiprai et al. similarly found no relationship between self-efficacy and a nursing professional’s age or years of nursing experience [23].

The results of simple descriptive statistical analysis showed that participants who engaged daily with SCS had higher self-confidence in SCS and more positive results for importance and beliefs and were more likely to practice SCS behaviors than participants who did not engage in SCS daily. This confirms that actual experience providing SCS in the course of one’s duties—for example, through outpatient smoking cessation services, health checkups, or preventive education—is an opportunity to master SCS skills and improve beliefs and perceived importance of SCS. These results also support the validity of analyzing the conceptual model for non-engagers and engagers separately. The results of structural equation modeling revealed that SCS self-efficacy and perceived importance of SCS, and beliefs were associated (covariance = 0.50) among participants who regularly engaged in SCS, while no such relationship was found among non-engagers. Thus, “experience” in one’s everyday duties serves as an opportunity to enhance “skills,” “importance and beliefs,” and other motivating factors. Guo et al. found that nurses’ attitudes as well as their frequency and practical experience in providing SCS, were significantly associated with psychiatric nurses’ self-efficacy toward SCS, a result that supports the findings of the present study [24].

The results of a simple descriptive statistical analysis of specific SCS behaviors (5A) were shown in Table 1. Except for Ask, engagers were implemented more than non-engagers. Past studies on implementation rates for the 5 As among nurses include a survey of 98 home care nurses (before SCS training) that found rates of 34.0% for Ask, 46.2% for Advise, 13.7% for Assess, 34.0% for Assist, and 0% for Arrange [21]; a study of 152 nurses in the Czech Republic that found rates of 63.0%, 45.8%, 38.9%, 26.0%, and 11.8%, respectively [14]; and a study of 507 nurses in Eastern Europe (before SCS training) reported rates of 70.4%, 65.7%, 58.6%, 36.3%, and 20.4%, respectively [25]. Li et al.’s study of 554 Japanese public health nurses using a 36-item survey on the 5 As found rates of 83.9%, 58.0%, 24.3%, 32.5%, and 43.9%, respectively [19]. These past findings, excluding those for home care nurses, indicate that nursing professionals Ask frequently but tend to have more difficulty with the Assess, Assist, and Arrange steps.

The results of the present study are similar to those of previous studies and suggest the particular importance of mastering the Assess, Assist, and Arrange steps if nursing professionals are to provide concrete SCS to their patients. The findings of the present study suggest that Assess, Assist, and Arrange are strongly influenced by SCS self-efficacy, beliefs, and perceived importance. A review by Carson et al. found that training medical professionals to practice SCS improved performance, particularly for Assist and Arrange, resulted in a drop in the prevalence of smoking-related diseases and led to higher cessation rates [26]. The findings of the present study indicate that promoting concrete SCS practices among nursing professionals requires that we strive to improve both self-efficacy and perceived importance of and beliefs about SCS.

The present study has some limitations. First, the survey did not ask about nursing professionals’ knowledge of SCS. Leung et al. indicated that specialized knowledge of smoking is an important independent predictor of a nurse’s provision of smoking cessation services [27], and studies have shown knowledge of smoking-related issues among Japanese nursing professionals to be quite low [28]. Thus, it is necessary to take into consideration the possibility that knowledge of smoking-related issues influenced the participants’ behavior in the present study. Second, the participants in the present study were nursing professionals who chose to participate in SCS workshops, and the results may therefore differ from the trends for all the nurses in Japan.

This study structurally presented the interrelationships among nursing professionals’ SCS self-efficacy, perceived importance, and beliefs explain their SCS behaviors. The results of this study showed that, in order for nurses to contribute to SCS, it is necessary for them to be convinced of the importance of SCS themselves, as well as to have self-efficacy toward SCS. It was also suggested that nurses who come in contact with many patients on a daily basis need to take the opportunity to provide support for smoking cessation on a daily basis. On the other hand, in a review of factors associated with smoking cessation support for nurses, four key concepts were identified as relevant: socioeconomic factors, smoking-related factors, motivational factors, and enabling factors and barriers [29]; this study was an investigation of some of these factors. In addition, there is a need for smoking cessation support for new tobacco products, such as the rise of heated cigarettes in Japan. The use of heated cigarettes has been publicly surveyed since 2018 in Japan, the 2019 results showed that about one-third of smokers use heated cigarettes [30]. There are studies demonstrating that nurses’ self-efficacy in supporting smoking cessation for new tobacco products is low [31]. In the future, educational interventions to support smoking cessation should be comprehensive, taking into account these social backgrounds. Paying attention not only to the negative aspect of smoking habit, but also to the broader aspect of the patient’s health habits, may be the perspective that nurses need to take in the future in order to promote new behavioral changes with patients [32].

## 5. Conclusions

Through structural analysis, this study showed the relationship between SCS behavior of Japanese nurses, self-efficacy toward SCS, and belief in and importance of SCS. The results suggested that the practice of SCS behavior is strongly influenced not only by self-efficacy for SCS but also by the belief in and importance of SCS. It is ideal for Japanese nurses to be able to perform SCS in various health care situations. Therefore, it is necessary to consider educational interventions that emphasize the importance of SCS.

## Figures and Tables

**Table 1:** Basic attributes.

		All Participants	(1) Engagers	(2) Non-Engagers	Comparison of (1) and (2)	
		*n* = 613	*n* = 266 (43.4)	*n* = 347 (56.6)	*p*-Value	Effect Size
Sex	Male	41 (6.7)	8 (3.0)	33 (9.5)	0.001	0.13
	Female	572 (93.3)	258 (97.0)	314 (90.5)		
Age		44.2 (SD 10.6)	44.6 (SD 9.6)	43.9 (SD 11.4)	0.757	0.01
Years as a nursing professional		19.6 (SD 10.3)	20.0 (SD 9.4)	19.3 (SD 11.0)	0.491	0.03
Smoking status	Current smoker	45 (7.3)	5 (1.9)	40 (11.5)	<0.001	0.19
	Ex-smoker	159 (25.9)	77 (28.9)	82 (23.6)		
	Never smoker	408 (66.6)	183 (68.8)	225 (64.8)		
	No response	1 (0.2)	1 (100)	0		
Type of nurses	Public health nurse	91 (14.8)	58 (21.8)	33 (9.5)	0.001	0.18
	Midwife	9 (1.5)	3 (1.1)	6 (1.7)		
	Nurse practitioner	489 (79.8)	193 (72.6)	296 (85.3)		
	Practical nurse	24 (3.9)	12 (50.0)	12 (3.5)		
Managerial position	Manager	178 (29.0)	63 (23.7)	115 (33.1)	0.011	0.10
	Non-manager	429 (70.0)	197 (74.1)	232 (66.9)		
	No response	6 (1.0)	6 (2.3)	0		
Type of facility	Hospital	441 (71.9)	157 (59.0)	284 (81.8)	<0.001	0.29
	Medical office/clinic	48 (7.8)	32 (12.0)	16 (4.6)		
	Health check-up center	38 (6.2)	30 (11.3)	8 (2.3)		
	Occupational health	25 (4.1)	17 (6.4)	8 (2.3)		
	Public health center	24 (3.9)	13 (4.9)	11 (3.2)		
	Other	37 (6.0)	17 (63.9)	20 (5.8)		
Status of SCS implementation (5 As)	1. Ask	402 (65.6)	169 (63.5)	233 (67.1)	0.660	0.04
(number of “always” or “usually” responses)	2. Advise	286 (46.7)	137 (51.5)	149 (42.9)	0.007	0.09
	3. Assess	211 (34.4)	119 (44.7)	92 (26.5)	<0.001	0.19
	4. Assist	121 (19.7)	86 (32.3)	35 (10.1)	<0.001	0.28
	5. Arrange	128 (20.9)	90 (33.8)	38 (11.0)	<0.001	0.28

Figures represent number of participants (%). (1)Engagers: Those who have the opportunity to engage in SCS on a daily work. (2) Non-engagers: Those who do not have the opportunity to engage in SCS on a daily work. Comparison of (1) and (2): Univariate analysis of engagers and non-engagers (Mann–Whitney *U* test used for age and years as a nursing professional; chi-squared test used for all other variables).

**Table 2:** SCS behaviors, perceived importance, attitude, and self-efficacy.

Item	Unit	All Participants	(1) Engagers	(2) Non-Engagers	Comparison of (1) and (2)	
		*n* = 613	*n* = 266	*n* = 347	*p*-Value	Effect Size
Behavior (α = 0.833)						
1. Ask (Confirm smoking status) [20]	5 choices	2.9 (1.1)	2.9 (1.0)	2.9 (1.2)	0.660	0.02
2. Advise (Encourage to quit smoking) [20]	5 choices	2.3 (1.1)	2.5 (1.1)	2.2 (1.2)	0.007	−0.11
3. Assess (Confirm stage) [20]	5 choices	1.9 (1.3)	2.3 (1.2)	1.5 (1.3)	<0.001	−0.30
4. Assist (Explain specific method) [20]	5 choices	1.4 (1.2)	2.0 (1.1)	1.0 (1.0)	<0.001	−0.44
5. Arrange (Refer to a specialist) [20]	5 choices	1.4 (1.2)	2.0 (1.1)	1.0 (1.1)	<0.001	−0.43
6. Extent to which the topic is discussed with patients [21]	4 choices	1.8 (0.8)	2.0 (0.9)	1.7 (0.8)	<0.001	−0.18
Self-efficacy (α = 0.739)						
7. Confidence in SCS counseling [21]	VAS	3.4 (2.2)	4.2 (2.1)	2.8 (2.0)	<0.001	0.32
8. Confidence in overall behavioral change counseling [21]	VAS	3.3 (2.1)	4.1 (2.1)	2.8 (1.9)	<0.001	0.30
9. Value of SCS counseling [21]	VAS	6.3 (2.8)	6.6 (2.6)	6.1 (2.9)	0.008	0.11
10. Importance of smoking cessation for patients or client [21]	VAS	8.3 (2.3)	8.4 (2.3)	8.2 (2.4)	0.646	0.02
Importance (α = 0.434)						
11. Extent of perceived importance of SCS at their organization [21]	5 choices	2.8 (1.0)	2.9 (0.9)	2.7 (1.0)	0.077	−0.07
12. Extent to which their patients or clients want to quit smoking [21]	5 choices	2.1 (1.0)	2.4 (1.0)	1.9 (1.0)	<0.001	−0.26
13. Extent to which their patients or clients believe that smoking is bad for their health [21]	5 choices	2.9 (1.0)	3.2 (0.8)	2.8 (1.0)	<0.001	−0.21
14. Asking patients or clients about smoking increases the chance of smoking cessation [12]	5 choices	2.4 (0.9)	2.7 (0.8)	2.2 (0.9)	<0.001	−0.25
15. Talking about smoking cessation with patients or clients improves relationships [12]	5 choices	2.2 (0.8)	2.4 (0.7)	2.1 (0.7)	<0.001	−0.15
16. Need to learn about SCS for their patients or clients [12]	5 choices	3.4 (0.6)	3.6 (0.5)	3.3 (0.6)	<0.001	−0.27
Beliefs (α = 0.598)						
17. Nursing professionals play an important role in patients’ SCS [12]	5 choices	3.1 (0.6)	3.2 (0.6)	3.0 (0.6)	<0.001	−0.20
18. Providing smoking cessation counseling to patients or clients is important [12]	5 choices	3.3 (0.6)	3.5 (0.6)	3.1 (0.7)	<0.001	−0.27
19. Smoking cessation counseling is time-consuming [12]	5 choices	2.4 (0.8)	2.4 (0.9)	2.5 (0.8)	0.214	0.05

Figures represent mean score (SD); α: Cronbach’s coefficient alpha. (1)Engagers: Those who have the opportunity to engage in SCS on a daily work. (2) Non-engagers: Those who do not have the opportunity to engage in SCS on a daily work. Comparison of (1) and (2): Univariate analysis of engagers and non-engagers (Mann–Whitney U test). Unit: Response format for each item. For questionnaire items 1–5, points were assigned as follows: Always = 5 points, Usually = 4 points, Sometimes = 3 points, Rarely = 2 points, and Never = 1 point. For questionnaire item 6, points were assigned as follows: Always = 4 points, Only when there is a connection to smoking = 3 points, Only when the patient brings it up = 2 points, and Never = 1 point. For questionnaire items 7–10, the number of points assigned corresponded to the VAS score of each item. For questionnaire items 11 through 19, points were assigned as follows: Strongly agree = 5 points, Agree = 4 points, Neither agree nor disagree = 3 points, Disagree somewhat = 2 points, and Disagree = 1 point.

**Table 3:** Exploratory factor analysis results.

Observed Variables	Pattern Coefficient				
	F1	F2	F3	F4	
	SCS Behaviors	SCS Self-Efficacy	Importance of SCS	Beliefs about SCS	
	α = 0.833	α = 0.479	α = 0.739	α = 0.634	
3. Assess	0.875	0.017	−0.052	−0.021	
2. Advise	0.772	0.076	0.044	−0.087	
4. Assist	0.710	−0.145	−0.037	0.079	
1. Ask	0.588	0.064	−0.030	−0.070	
5. Arrange	0.584	−0.093	0.035	0.118	
6. Extent to which the topic is discussed with patients	0.444	0.005	0.089	0.032	
7. Confidence in SCS counseling	0.037	0.980	0.009	0.037	
8. Confidence in overall behavioral change counseling	0.010	0.892	0.013	0.041	
9. Value of SCS counseling	−0.050	0.280	−0.172	−0.112	*
19. Smoking cessation counseling is time-consuming	−0.018	0.175	0.085	−0.020	*
Years as a nursing professional	0.046	0.170	−0.024	−0.096	*
16. Need to learn about SCS for their patients or clients	0.032	0.129	0.837	−0.017	
18. Providing smoking cessation counseling to patients or clients is important	−0.008	0.011	0.788	−0.036	
17. Nursing professionals play an important role in patients’ SCS	−0.032	−0.065	0.631	0.047	
10. Importance of smoking cessation for patients or clients	−0.051	0.000	−0.230	−0.162	*
12. Extent to which their patients or clients want to quit smoking	−0.036	0.025	−0.089	0.910	
13. Extent to which their patients or clients believe that smoking is bad for their health	−0.032	0.006	0.014	0.603	
15. Talking about smoking cessation with patients or clients improves relationships	−0.070	−0.062	0.119	0.364	
11. Extent of perceived importance of SCS at their organization	0.121	0.046	0.047	0.265	*
14. Asking patients or clients about smoking increases the chance of smoking cessation	0.039	−0.077	0.222	0.234	*
Factor contribution	4.23	3.33	3.2	2.98	
Cumulative contribution rate	23.27	31.46	37.02	41.33	

Promax oblique rotation, maximum likelihood method; * Factor loading of 0.3 or below. F1: Factor 1, F2: Factor 2, F3: Factor 3, and F4: Factor 4. α: Cronbach’s coefficient alpha.

## Data Availability

All data generated or analyzed during this study are included in this published article. The data of the questionnaire performed in this study are available from the authors.
